# Feasibility of Augmented Reality in Orthopedic Trauma Care: A Narrative Review of Current Applications

**DOI:** 10.7759/cureus.92088

**Published:** 2025-09-11

**Authors:** Eyad Z Mansour, Augustine M Saiz

**Affiliations:** 1 Medicine, California Health Sciences University College of Osteopathic Medicine, Clovis, USA; 2 Orthopaedic Surgery, UC Davis Health, Sacramento, USA

**Keywords:** augmented reality, head-mounted device, orthopedic surgery, robotic navigation, trauma

## Abstract

Augmented reality (AR) is an emerging technological aid for surgeons, offering enhanced visualization, improved surgical precision, reduced surgical radiation exposure, and real-time access to patient anatomical data. AR is currently being explored for its role in surgical care, but its role in orthopedic trauma care remains undefined. Recent investigations include fracture fixation using intraoperative navigation, surgical training, and education. Despite promising results from early studies, the lack of standardized literature and protocols has limited the adoption of AR devices. In this review, we will explore the current applications and practicality of AR in the management of orthopedic trauma cases, highlighting its potential and limitations.

## Introduction and background

Augmented reality (AR) is a technology that overlays images and data with the real-world environment, thereby transforming the way surgeons interact with patient images and planned surgical interventions. Typically, AR utilizes a head-mounted device (HMD) to enhance a surgeon’s experience.

Currently, AR-HMDs are being investigated in the surgical, educational, and physical therapy settings [1-5]. In orthopedics, AR displays the ability to enhance the accuracy of complex procedures, reduce radiation, and improve education [6-13]. However, across the domain of orthopedics, AR has been limited by the lack of clinical studies and absence of standardization [1,13]. 

The gap is particularly evident in orthopedic trauma care, where unpredictable and acute cases often occur. Fracture fixation is a common trauma procedure performed in both young and elderly patients with varying degrees of bone and soft tissue damage that occur in the long bones, the pelvis, or periarticular regions [14-16]. AR offers the potential to enhance surgical precision, improve fracture reduction accuracy, and optimize implant placement, potentially leading to improved patient outcomes and reduced complication rates [1,2]. This may be especially relevant for less experienced and young surgeons.

The primary objective of this narrative review is to summarize the existing evidence of AR-HMDs in orthopedic trauma care, their potential benefits, and their limitations. This review aims to guide future research efforts on the use of AR in orthopedic care and education, ultimately translating into clinical practice.

## Review

Methods

Search Strategy and Selection Criteria

A comprehensive search of the electronic database PubMed was performed using a combination of keywords for the literature search: augmented reality, orthopedic trauma, fracture fixation, surgical navigation, surgery, and 3D-HMD. The search was performed from May 2025 to June 2025.

Due to the limited research in orthopedic trauma, the inclusion criteria encompassed studies in related subspecialties of orthopedic surgery, such as total joint arthroplasty, hip, shoulder, knee, and spine surgery. Clinical, cadaveric, and synthetic-based studies that reported quantitative data, qualitative data, and technical outcomes relating to the precision and accuracy of AR were included. Case reports, randomized controlled trials, case-control studies, clinical trials, and case series were inspected for inclusion. Studies were excluded if they did not involve orthopedic procedures, did not involve AR technology, were not written in English, or if the methods and outcomes were not clearly defined.

**Figure 1:**
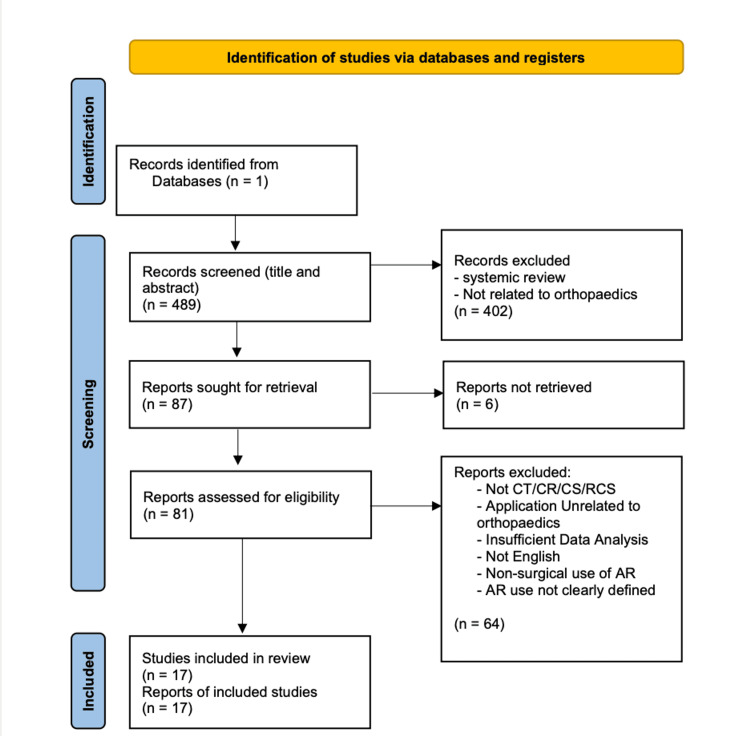
Adapted PRISMA Study Identification and Selection Flow Diagram CT: Clinical Trial; CR: Case Report; CS: Case Series; RCS: Randomized Control Study; PRISMA: Preferred Reporting Items for Systematic Reviews and Meta-Analyses

This narrative review adapted a flow diagram from Preferred Reporting Items for Systematic Reviews and Meta-Analyses (PRISMA) guidelines to provide transparency in the search strategy and screening process (Figure 1) [17].

Study Selection

Four hundred and eighty-nine potential studies were inspected by title and abstract, but 81 were eventually assessed for eligibility. Ultimately, 17 studies met the inclusion criteria and were selected for this review.

Data Extraction

Data extraction was performed independently by one reviewer, who recorded information from each study that met inclusion standards in Microsoft Excel 365 (Redmond, Washington, USA), including study design, patient population, orthopedic procedure, AR technology used, outcome measures, and reported complications. 

To ensure consistency and accuracy, any discrepancies between the studies were resolved through consensus discussion.

Current evidence

Seventeen studies were selected based on addressing the role of AR in orthopedic care; six were case reports/series (35.3%), three were clinical trials or randomized control trials (RCTs) (17.6%), and eight were cadaver- and/or synthetic-based studies (47.1%) (Table 1).

**Table 1:** Summary of Published Studies, the Type of the Study That Was Performed, the Equipment and Function AR Served During the Study, and the Joint of Interest AR: Augmented Reality; HMD: Head-Mounted Device

Study	Type	System	Joint
Park et al. [18]	Case Report	Apple Pro Vision Headset; Displaying EMR and MRI images,	Spine
Umebayashi et al. [19]	Case Report	AR-HMD assisted intraoperative navigation and imaging	Spine
Cofano et al. [20]	Case Series	Epson BT-300, Epson BT-350, Vzix, Microsoft Holo Lens HMDs; Surgical Planning (3D model), Telemonitoring, Teaching	Spine
Rojas et al. [21]	Case Series	AR-HMD 3D Surgical Planning (3D model) and intraoperative navigation	Shoulder
Legosz et al. [22]	Case Report	AR-HMD, 3D overlay of anatomical structures	Hip
Leal et al. [23]	Case Report	AR-HMD, 3D overlay of anatomical images and intraoperative navigation	Hip
Bischofreiter et al. [24]	Clinical Trial	ARIN System (NexTAR, Medacta International); AR-HMD 3D guidance and intraoperative navigation	Shoulder
Ma et al. [25]	RCT	Microsoft HoloLens; navigation system developed by Shanghai Linyan Medical Technology Co. Ltd.; optical tracking system (NDI, Polaris Vega, Canada); 3D intraoperative navigation and overlay of of anatomical images	Spine
Tanino et al. [26]	RCT	Smart Phone based AR Navigation (AR-Hip, Zimmer Biomet Japan	Hip
Klopfer et al. [27]	Synthetic Study	Microsoft HoloLens2 AR-HMD; 3D guidance and anatomical images	Tibia
Hazra et al. [28]	Cadaveric Study	ARIN System (NexTAR, Medacta International); AR-HMD 3D guidance and intraoperative navigation	Shoulder
Rojas et al. [29]	Cadaveric Study	AR-HMD 3D Surgical Planning (3D model) and intraoperative navigation	Shoulder
Iqbal et al. [30]	Pilot Study	Microsoft Hololens; Surgical robot (NAVIO, Smith and Nephew plc.); 3D Intraoperative guidance and user-interface of the surgical robot displayed	Knee
Burström et al. [31]	Cadaveric Study	AR-HMD 3D Surgical Planning (3D model) and intraoperative navigation	Spine
Huang et al. [32]	Cadaveric Study	AR radiograph superimposition; real-time navigation and tracking	Spine
Wang et al. [33]	Cadaveric Study	AR-HMD 3D Surgical Planning (3D model) and intraoperative navigation	Hip
Molina et al. [34]	Cadaveric Study	AR-HMD (XVision, Augmedics Ltd.);3D Surgical Planning (3D model) and intraoperative navigation	Spine

Case Reports and Case Series

Various case reports and case series have provided evidence for AR's feasibility in clinical practice. Park et al. reported on an 84-year-old male who was diagnosed with L4-5 spinal stenosis via MRI. A unilateral laminotomy and bilateral decompression were performed using an AR-HMD displaying MRI and EMR data in real time and the procedure was performed without “difficulty or complications” [18]. Similarly, a dual case report was performed on patients with cervical spine pain. One patient underwent an anterior cervical discectomy and transvertebral anterior cervical foraminotomy. The other underwent a laminoplasty and posterior cervical foraminotomy. Both procedures were performed with an AR-HMD that displayed preoperatively planned navigation. Post-operative CT scans displayed that the surgeries were performed with accuracy similar to the navigation system [19].

Furthermore, a case series of 12 lumbar arthrodesis surgeries was performed with an AR-HMD using navigation and preoperatively planned data. All the surgeries were performed with accuracy relative to planned screw trajectories with no complications related to the AR device. The surgeons self-reported positive feedback via a questionnaire [20]. Rojas et al. also utilized real-time preoperatively planned data and CTs, which were displayed on the AR-HMD during reverse total shoulder arthroplasty (RTSA) in seventeen patients. This case series compared intra- and postoperative values and pre- and postoperative angle values for the glenoid component placement and found that all mean deviations were within the range of acceptable limits, and placement time was comparable to traditional methods [21].

Interestingly, in the first case report for a custom-revision hip arthroplasty using an AR-HMD, implant fixation was performed successfully, and early positive outcomes from the patient were observed [22]. In a similar report, AR navigation was successfully used to perform a successful revision conversion from hip fusion to a total hip arthroplasty, and after one year, pain was reported to have subsided, and the patient's function improved [23].

Clinical Trials and RCTs

One clinical trial utilized AR-HMD to perform 20 RTSA procedures to investigate the learning curve of AR intraoperative navigation. Surgery time (P = 0.001) and blood loss decreased (0.005). It should be noted that significant changes in surgery time and blood loss came in the 14th and 15th surgeries, respectively [24].

Ma et al. evaluated AR-guided pedicle screw fixation (75 control; 71 experimental). Screw placement for the experimental group was 16.33 ± 9.93 minutes, and that the control group was 30.32 ± 10.40 minutes (P < 0.05). Total surgical time was 180.73 ± 47.37 minutes in the AR group and 169.89 ± 65.05 minutes in the control group (P < 0.05). The AR group had 344 out of 351 (98%) screw placements rated as excellent, while the control group only had 319 out of 344 (92.7%) (P = 0.007). Finally, no complications were associated with the AR-HMD [25].

A novel approach was used with a smartphone-based AR navigation system for hip implant cup positioning in total hip arthroplasty. AR had 34 of 36 (94%) cups within the Lewinnek safe zone, and the control group had 23 of 36 cups (64%), respectively (P < 0.001). AR achieved desired cup positioning with a median 1° deviation from desired placement, and the control group had a median 5° deviation (P = 0.001). No differences were observed in operation time and Hip Disability and Osteoarthritis Outcome Scores [26].

Cadaveric and Synthetic-Based Studies

Klopfer et al. reported successful tibial nail implantation in their synthetic models, and the surgeons reported that AR displayed useful data during the surgery [27]. Hazra et al. found decreased differences in planned vs. obtained inclination when using AR for RTSA in cadavers [28]. Similarly, in RTSA in cadavers, no significant deviation was found for mean planned vs. postoperative values with 1.0° ± 0.7° inclination, 1.8° ± 1.3° retroversion, 1.1 ± 0.4 mm entry point, 0.7 ± 0.6 mm depth, and 1.7° ± 1.6° rotation [29].

Iqbal et al. examined 10 surgeons performing a robotic-assisted patellofemoral arthroplasty on a synthetic knee using AR enhanced with a robotic user interface and compared it with conventional techniques. Similar outcomes were observed relative to cutting time with a mean difference of 6.111 seconds (P = 0.407) and surface roughness (P = 0.274). No negatives were reported from using the AR, and surgeon feedback was positive in terms of usability, lack of neck/eye strain, and openness for adoption in a postoperative survey [30].

Two studies utilized animal cadavers to perform minimally invasive spine surgery [31,32]. Using AR-HMD displaying preoperative scans and planned navigation software, Burström et al. found no significant differences in pre-planned vs postoperative screw insertion values with a mean deviation of 1.7° ± 1.7° in the axial plane and 1.6° ± 1.2° in the sagittal plane [31]. Similarly, using AR-HMD superimposed imaging, navigation, and a real-time tracking system, Huang et al. found no significant difference in mean deviations of preplanned vs postoperative anteroposterior and lateral screw insertion with 0.74 ± 0.21 mm and 1.13 ± 0.40 mm, respectively, and anteroposterior and lateral puncture needle depth with 1.26 ± 0.20 mm and 1.22 ± 0.25 mm, respectively [32].

Two cadaveric studies examined the use of AR-assisted spinal screw surgical implantation. All screws were inserted with high accuracy relative to planned trajectories [33,34]. Mean deviations in sacroiliac screws of planned vs. inserted were 2.7 ± 1.2 mm at bony entry and 3.7 ± 1.1 mm at screw tip, and mean angular deviations were 2.9° ± 1.1° [33]. Molina et al. had each screw placement graded by neuroradiologists and found accuracy > 94% for 120 screws [34].

Discussion

Looking into the future, AR-HMD systems do appear to be potentially transformative for orthopedic trauma cases, offering improved precision and enhanced training benefits despite limited direct evidence. AR-HMDs allow surgeons to view patients' scans, preplanned data, and utilize intraoperative navigation as a virtual overlay without having to maneuver head and eye orientation away from the patient. Currently, our understanding of AR is still unclear compared to traditional methods. More clinical testing and standardization of training are required before utilization in orthopedic trauma cases.

Surgical Accuracy and Navigation

Multiple trials across complex spine, shoulder, and hip procedures showed acceptable navigational accuracy with AR guidance, reduced deviation from planned trajectories, and higher rates of optimal placement zones [25-34]. Applying these outcomes to trauma cases, where fracture alignment is critical, suggests that AR could substantially aid in accurate fracture reduction and implant positioning. In human-based case reports/series, AR didn’t appear to cause any complications, and all surgeries were performed successfully with accuracy [18-23]. Longitudinal studies are required to confirm if any drawbacks in accuracy and surgical outcomes occur from the use of AR-HMDs.

Radiation

Radiation reduction is a key advantage of AR in orthopedic settings, particularly in trauma cases involving extensive fluoroscopy. The use of AR-navigated procedures could potentially reduce the use of fluoroscopy in operating rooms. However, it should be noted that radiation exposure is less problematic when personal protection equipment is used [35-38].

Limitations and Workflow Integration

Despite the benefits and innovation of AR, challenges remain. AR systems often result in increased setup time and require training and have a learning curve-factors that may hinder utility in urgent trauma settings [24]. The current lack of standardized AR protocols for surgery further complicates clinical adoption [13,39]. Furthermore, AR faces ethical, legal, and patient consent-related pitfalls [40]. Standards and protocols will most likely be needed to ensure patient safety and proper usage of AR-HMDs before the adoption of this technology occurs.

Future Applications

AR may have potential use for teaching and developing the skills of young orthopedic trauma surgeons to refine speed and procedural efficiency [1,2]. This could enhance the learning experience for young surgeons and residents, allowing them to participate and view surgical procedures in real time. With orthopedic trauma cases being vastly unpredictable and acute, the current literature directs us towards further development and research to better understand the efficacy of AR-HMD in unpredictable and acute scenarios.

## Conclusions

Promising data from related orthopedic fields emphasize the limited research available in orthopedic trauma care. AR shows significant potential to enhance accuracy, improve surgical navigation, and support education. However, vigorous clinical data and standardized protocols are necessary to fully establish AR’s role in the response to acute trauma settings.
